# Enhancing Thermoelectric Properties of (Cu_2_Te)_1−*x*_-(BiCuTeO)*_x_* Composites by Optimizing Carrier Concentration

**DOI:** 10.3390/ma15062096

**Published:** 2022-03-11

**Authors:** Wenyu Zhang, Zhifang Zhou, Yueyang Yang, Yunpeng Zheng, Yushuai Xu, Mingchu Zou, Ce-Wen Nan, Yuan-Hua Lin

**Affiliations:** State Key Laboratory of New Ceramics and Fine Processing, School of Materials Science and Engineering, Tsinghua University, Beijing 100084, China; zhang-wy20@mails.tsinghua.edu.cn (W.Z.); zhouzf@mail.tsinghua.edu.cn (Z.Z.); yangyuey19@mails.tsinghua.edu.cn (Y.Y.); zhengyp15@tsinghua.org.cn (Y.Z.); xuyushuai5736@163.com (Y.X.); zoumc@mail.tsinghua.edu.cn (M.Z.); cwnan@mail.tsinghua.edu.cn (C.-W.N.)

**Keywords:** thermoelectric materials, Cu_2_Te-BiCuTeO composites, carrier concentration

## Abstract

Because of the high carrier concentration, copper telluride (Cu_2_Te) has a relatively low Seebeck coefficient and high thermal conductivity, which are not good for its thermoelectric performance. To simultaneously optimize carrier concentration, lower thermal conductivity and improve the stability, BiCuTeO, an oxygen containing compound with lower carrier concentration, is in situ formed in Cu_2_Te by a method of combining self-propagating high-temperature synthesis (SHS) with spark plasma sintering (SPS). With the incorporation of BiCuTeO, the carrier concentration decreased from 8.1 × 10^20^ to 3.8 × 10^20^ cm^−3^, bringing the increase of power factor from ~1.91 to ~2.97 μW cm^−1^ K^−2^ at normal temperature. At the same time, thermal conductivity reduced from 2.61 to 1.48 W m^−1^ K^−1^ at 623 K. Consequently, (Cu_2_Te)_0.95_-(BiCuTeO)_0.05_ composite sample reached a relatively high *ZT* value of 0.13 at 723 K, which is 41% higher than that of Cu_2_Te.

## 1. Introduction

With the capability of direct conversion from thermal energy to electrical energy through the movement of internal phonons and charge carriers, thermoelectric (TE) materials are widely researched and used in cogeneration, electronic refrigeration and thermal sensors [1,2,3,4,5,6,7]. Thus, TE materials play an increasingly crucial part in sustainable development. The energy conversion efficiency of a TE material is mainly depicted by its dimensionless figure of merit $ZT=\sigma S^{2}T/\kappa$, where σ, S, T and κ are the electrical conductivity, Seebeck coefficient, absolute temperature and thermal conductivity, respectively [8,9]. Therefore, TE materials with high power factor (PF), which is defined as $\mathrm{PF}=\sigma S^{2}$, but low thermal conductivity, are suitable for thermoelectric applications. To get the ideal thermoelectric performance, the PF of materials can be raised by band engineering, quantum confinement effects and electron energy barrier filtering [10,11,12,13,14,15,16]. Besides, κ can be reduced by nanostructures all-scale hierarchical architecture engineering, which is able to increase the scattering of short, medium and long wavelength phonons, and entropy engineering [17,18,19,20,21,22].

Among the TE materials, transition-metal chalcogenides, especially tellurides, such as PbTe [17,23], Bi_2_Te_3_ [24,25] and AgSbTe_2_ [26,27], have been widely studied because of their high carrier mobilities and remarkably low thermal conductivities. In recent years, copper telluride (Cu_2_Te) [28,29], along with copper selenide (Cu_2_Se) [30] and copper sulfide (Cu_2_S) [31], have emerged as promising thermoelectric materials. This kind of material has exhibited great thermoelectric performance due to its low thermal conductivity and electrical resistivity. Compared to Cu_2_Se and Cu_2_S, Cu_2_Te should have more appealing thermoelectric performance owing to the lower ionicity of chemical bonds between Cu and Te due to the lower electronegativity of Te in comparison to Se or S, which is beneficial for obtaining large carrier mobility (*μ*) and improving its carrier conductivity. Besides, the lattice thermal conductivity (*κ*_L_) of Cu_2_Te is expected to be lower than that of Cu_2_S and Cu_2_Se because tellurium is much heavier than sulfur and selenium. These two points make Cu_2_Te a theoretical potential TE material with high *ZT* [32]. However, an abnormality has been found ascribed to two aspects. One of them is that the hole concentration (pH) of Cu_2_Te is too high because of its severe copper deficiency, which easily forms nonstoichiometric Cu_2-δ_Te compound [33]. The other is that the phase diagram of Cu_2_Te is too complex, especially, it has at least five phase transitions from room temperature to its melting point [34].

Several researches have been made to improve the thermoelectric property of Cu_2_Te in recent years. Dabin Park, et al. [28] synthesized Te/Cu_2_Te nanorod composites with 1D nanostructure through a solution phase mixing process by using Cu_2_Te with different properties and polyvinylpyrrolidone (PVP) in 2018. Sayan Sarkar, et al. [29] synthesized Ga-doped Cu_2_Te, Cu_1.97_Ga_0.03_Te, to improve the electrical conductivity and Seebeck coefficients of Cu_2_Te in 2019. However, the way of incorporating with oxide to optimize the thermoelectric properties of Cu_2_Te is rarely reported.

BiCuXO (X = S, Se and Te) oxychalcogenides have a layered structure that is made up of (Bi_2_O_2_)^2+^ insulating layers alternately stacked with (Cu_2_X_2_)^2^^−^ conductive layers as a charge reservoir along the *c*-axis [35,36,37]. With regard to the thermoelectric properties of BiCuXO, most previous studies focus on BiCuSeO due to the high Seebeck coefficient and low thermal conductivity, but low conductivity [38]. Besides, oxide-containing compounds have better chemical and thermal stability [39]. Based on the above, we planned to combine Cu_2_Te with BiCuTeO to lower the thermal conductivity and improve the Seebeck coefficient of Cu_2_Te by optimizing the carrier concentration.

Herein, we synthesized (Cu_2_Te)_1−x_-(BiCuTeO)*_x_* composites by self-propagating high-temperature synthesis (SHS) and spark plasma sintering (SPS) process to optimize carrier concentration (*n*) and decrease the total thermal conductivity. As a result, the PF was raised from ~1.91 to ~2.97 μW cm^−1^ K^−2^ at normal temperature, and the (Cu_2_Te)_0.95_-(BiCuTeO)_0.05_ composite sample reached a relatively high *ZT* value of 0.13 at 723 K, which is 41% higher than that of pristine Cu_2_Te.

## 2. Materials and Methods

(Cu_2_Te)_1−x_-(BiCuTeO)*_x_* (*x* = 0, 0.05, 0.1, 0.2, 0.3, 0.4) samples were prepared by self-propagating high-temperature synthesis (SHS) and spark plasma sintering (SPS) process. Commercial high-purity powders of Bi (4N, Innochem, Meerhout, Belgium), Cu (AR, Meryer, Shanghai, China), Te (4N, Aladdin, Shanghai, China) and Bi_2_O_3_ (3N, Meryer) were weighed with a stoichiometry of (Cu_2_Te)_1−x_-(BiCuTeO)*_x_* (*x* = 0, 0.05, 0.1, 0.2, 0.3, 0.4), and then mixed uniformly in an agate mortar in air. The mixed powders were cold-pressed into a pellet using a steel die under 6 MPa in air and the bulk was put in an alumina crucible. The SHS process was started by heating the bottom of the crucible to the ignition temperature with an alcohol lamp in air. Once the reaction began, we put the lid on the cauldron and moved the alcohol lamp away. The combustion wave was persisted by the energy released by the initial reaction and it spread to the whole bulk in a few seconds. The product after SHS was crushed into powders and the obtained powders were densified by SPS furnace (Sumitomo SPS-1050T, Fuji, Japan) for 10 min at 773 K under axial pressure of 40 MPa.

The micro-structure of all prepared bulks was characterized by the X-ray diffraction (XRD; D/max-2500/PC, Rigaku, Japan). The fractured surface morphology of the samples was studied by field emission scanning electron microscopy (FESEM; ZEISS-MERLIN, Germany), equipped with energy dispersive spectrometer (EDS). The morphology and chemical composition of the nano-powders (prepared by grounding bulks into powders and ball milling following by ultrasonic treatment into nano-powders) were characterized by high-resolution transmission electron microscopy (TEM; JEM-2100F, JEOL, Tokyo, Japan) with an integrated energy dispersive spectrometer (EDS), and the corresponding fast Fourier transform (FFT) patterns are obtained by RADIUS software. The electrical conductivity (*σ*) and Seebeck coefficient (*S*) were simultaneously measured by using a ZEM-3 instrument (ULVAC-RIKO, Yokohama, Japan). Thermal conductivity (*κ*) was measured indirectly according to the equation $\kappa =DC_{P}\rho$, where *D* is thermal diffusivity as measured by the LFA 457 MicroFlash (Netzsch, Weimar, Germany), ρ is the mass density of samples determined according to the Archimedes method and $C_{P}$ is the specific heat capacity calculated according to the Neumann–Kopp rule [40]. The Hall coefficients were investigated at room temperature and a magnetic field of 0.7 T, from which the carrier concentration and mobility of all samples was able to be determined.

## 3. Results and Discussion

**Figure 1:**
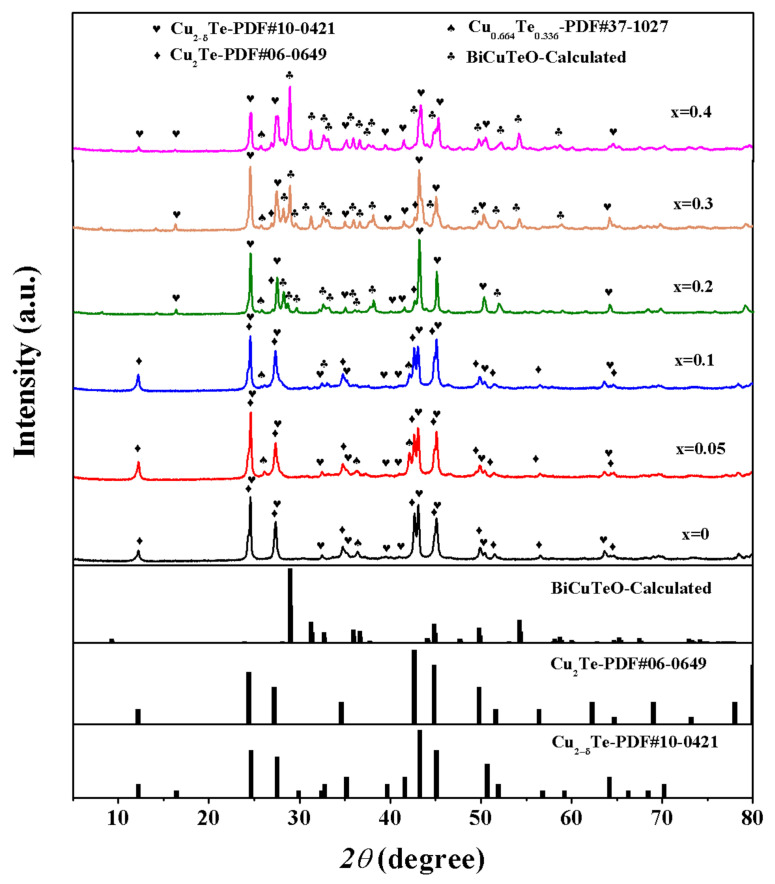
Bulk XRD patterns for (Cu_2_Te)_1−x_-(BiCuTeO)*_x_* (*x* = 0, 0.05, 0.1, 0.2, 0.3, 0.4).

Cu_2_Te, along with its nonstoichiometric compound Cu_2-δ_Te, has a very complicated crystal structure among all the copper chalcogenides [33]. The crystal structure at room-temperature for prepared (Cu_2_Te)_1−x_-(BiCuTeO)*_x_* bulk samples, as presented in Figure 1, is very complicated as well, and consists of different phases. As *x* = 0, the Cu_2_Te is composed of Cu_2_Te (PDF#06-0649), Cu_2−δ_Te (PDF#10-0421) and Cu_0.664_Te_0.336_ (PDF#37-1027), similar to the previous study [33]. When *x* = 0.1, the peaks of BiCuTeO are detected at 33°, and become more and more pronounced with the content of BiCuTeO growing. Since *x* = 0.2, the peaks of Cu_2_Te (PDF#06-0649) start to decrease, because the Cu and Te reacted with Bi and O to form BiCuTeO. The standard Powder Diffraction File (PDF) card is calculated according to the literature data [41].

**Figure 2:**
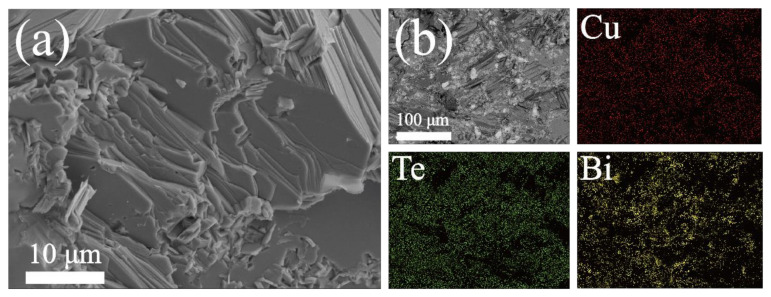
(**a**) FESEM fractography for (Cu_2_Te)_0.9_-(BiCuTeO)_0.1_; (**b**) EDS for (Cu_2_Te)_0.9_-(BiCuTeO)_0.1_.

To prove structure evolution of the (Cu_2_Te)_1−x_-(BiCuTeO)*_x_* and the in situ formation of BiCuTeO, detailed microscopic investigations were made on the prepared Cu_2_Te samples. Two phases with obviously different contrast were presented in the cross-sectional microstructures of (Cu_2_Te)_1−x_-(BiCuTeO)*_x_* shown in Figure 2a,b. It can be easily seen that there are a large number of tiny dispersive particles homogeneously clung or embedded to each layer. Due to the intrinsic layered feature of Cu_2_Te, and the gathering of Bi which was revealed in Figure 2b through the energy dispersive spectrometer (EDS), the powders with lower contrast should be BiCuTeO and the slices with higher contrast are Cu_2_Te.

**Figure 3:**
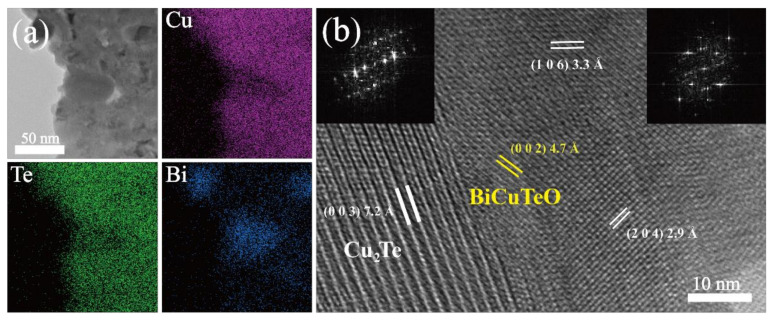
(**a**) TEM and EDS for (Cu_2_Te)_0.9_-(BiCuTeO)_0.1_. (**b**) HRTEM for (Cu_2_Te)_0.9_-(BiCuTeO)_0.1_. The insets are the corresponding fast Fourier transform (FFT) patterns for the areas marked in Figure 3b.

TEM investigation was performed on (Cu_2_Te)_0.9_-(BiCuTeO)_0.1_ sample to research microstructure information, as revealed in Figure 3a,b. A few tinier nanocrystals of 50 nm could be observed in Figure 3a and was proved to be bismuth content clumping together by EDS analysis. Figure 3b shows the HRTEM image of (Cu_2_Te)_0.9_-(BiCuTeO)_0.1_. The lattice fringe space measured as 4.7 Å may correspond to the (002) plane of BiCuTeO, while differently oriented lattice spacing of about 7.2 Å, 3.3 Å and 2.9 Å may correspond to (003), (106) and (204) planes of Cu_2−δ_Te, respectively. Actually, the lattice spacing of about 7.2 Å and 3.3 Å could also be assigned to the (031) and (162) planes of Cu_2_Te, because of their similar crystal structure.

**Figure 4:**
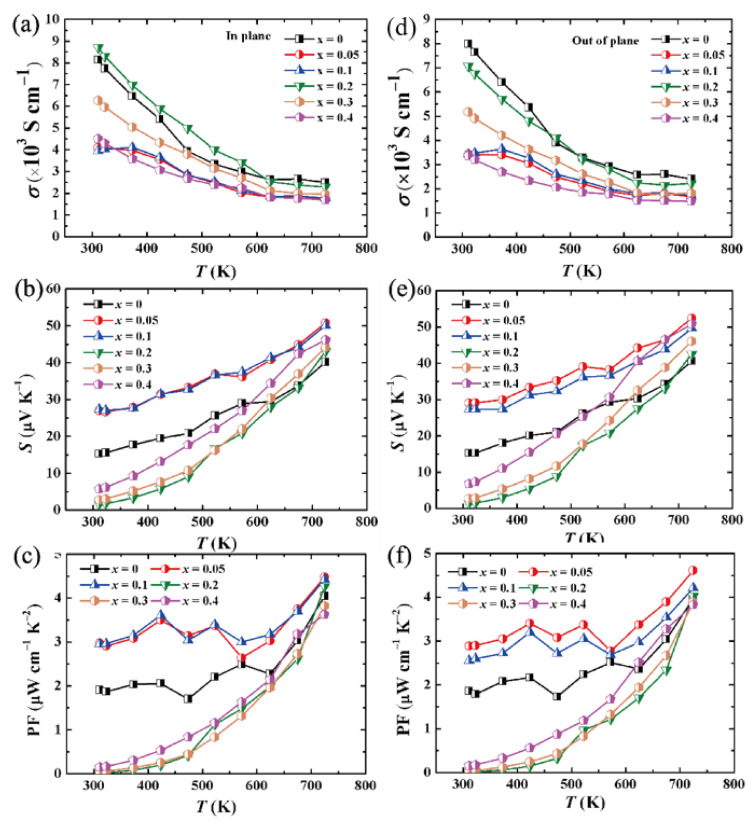
In plane electrical properties of (Cu_2_Te)_1−x_-(BiCuTeO)*_x_*: (**a**) electrical conductivity, (**b**) Seebeck coefficient, (**c**) power factor. Electrical properties of (Cu_2_Te)_1−x_-(BiCuTeO)*_x_*; out-of-plane electrical properties of (Cu_2_Te)_1−x_-(BiCuTeO)*_x_*: (**d**) electrical conductivity, (**e**) Seebeck coefficient, (**f**) PF.

Since Cu_2_Te has a complex phase diagram containing at least five phase transitions from room temperature to its melting point [34], the electrical performance of Cu_2_Te is abnormal with temperature change. Figure 4 displays the electrical properties of (Cu_2_Te)_1−x_-(BiCuTeO)*_x_* as a function of temperature, where (a)–(c) are the in plane electrical conductivity, Seebeck coefficient and power factor, respectively. As presented in Figure 4a, the electrical conductivity reduces except for the phase transition regions as the temperature increases, behaving as a highly degenerate semiconductor. When *x* is less than 0.1 and more than 0.2, the electrical conductivity is decreased as the content of BiCuTeO increases, while the electrical conductivity increases from *x* = 0.1 to *x* = 0.2. The reason should be ascribed to the variable carrier concentration and mobility, as demonstrated in Table 1. Since *x* = 0.2, Cu_2_Te (PDF#06-0649), one of the main phases, begins to decrease until it almost disappears, while the phase of BiCuTeO increases. That leads to the decrease of the carrier concentration and increase of the carrier mobility, as Cu_2_Te has a high carrier concentration and BiCuTeO has a low carrier concentration but high mobility.

All the samples display p-type conducting within the measured temperature range on account of the intrinsic copper deficiencies in Cu_2_Te, as shown in Figure 4b, which is proved by the positive Seebeck coefficient. The Seebeck coefficient for all samples is relatively low, even no more than 60 μV K^−1^ at 723 K. Such a low Seebeck coefficient is related to the high carrier concentration. Among all the samples, (Cu_2_Te)_0.95_-(BiCuTeO)_0.05_ and (Cu_2_Te)_0.9_-(BiCuTeO)_0.1_ composites show the highest Seebeck coefficient values of 26.94 μV K^−1^ and 27.33 μV K^−1^ at room temperature, respectively. Besides the appropriate carrier concentration, the DOS effective mass (m*) should be another reason. Thus, it is necessary to calculate the m*. The calculations are based on assumption of acoustic phonon scattering and the single parabolic band (SPB) mode [19]. Although the calculation of a single parabolic band model in a complex system is not that accurate, especially after the content of BiCuTeO has increased, a trend could be observed according to the results. It can be seen from Table 1, that (Cu_2_Te)_0.95_-(BiCuTeO)_0.05_ and (Cu_2_Te)_0.9_-(BiCuTeO)_0.1_ composites have the highest m* of 0.785 *m*_0_ and 0.716 *m*_0_. The PF of (Cu_2_Te)_1−x_-(BiCuTeO)*_x_* as a function of temperature is displayed in Figure 4c. The variation in PF is very similar to that in Figure 4b, illustrating that Seebeck coefficient plays a decisive role in altering the PF of (Cu_2_Te)_1−x_-(BiCuTeO)*_x_*. The PF of pristine Cu_2_Te is 4.05 μW cm^−1^ K^−2^ at 723 K, and the PF of (Cu_2_Te)_0.95_-(BiCuTeO)_0.05_ and (Cu_2_Te)_0.9_-(BiCuTeO)_0.1_ composites increase to 4.49 μW cm^−1^ K^−2^ and 4.43 μW cm^−1^ K^−2^ at 723 K, respectively. The difference of PF at high temperature is not so obvious, but the improvement of PF is large at room temperature. The PF increased from 1.91 (for pristine Cu_2_Te) to 2.97 μW cm^−1^ K^−2^ (for the (Cu_2_Te)_0.95_-(BiCuTeO)_0.05_) at normal temperature. Figure 4d–f display the out of plane electrical conductivity, Seebeck coefficient and power factor, and are similar to Figure 4a–c, indicating that (Cu_2_Te)_1−x_-(BiCuTeO)*_x_* composites have nearly isotropic transports.

**Figure 5:**
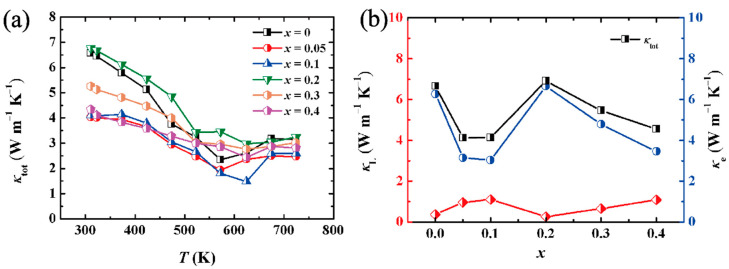
(**a**) Thermal conductivity of (Cu_2_Te)_1−x_-(BiCuTeO)*_x_* as a function of temperature. (**b**) Lattice thermal conductivity (*κ*_L_) and carrier thermal conductivity (*κ*_e_) as a function of BiCuTeO content at room temperature.

The total thermal conductivity (*κ*_tot_) of (Cu_2_Te)_1−x_-(BiCuTeO)*_x_* composites shown in Figure 5a is quite high, which are due to the inherent metallic property of Cu_2_Te and the high hole concentration caused by unavoidable deviation from stoichiometry. They show complicated temperature dependencies in the temperature ranged from 300 to 723 K, first decreasing and then increasing as a whole over the entire temperature range, which is the effect of the phase transition of Cu_2_Te [29]. (Cu_2_Te)_0.95_-(BiCuTeO)_0.05_ and (Cu_2_Te)_0.9_-(BiCuTeO)_0.1_ composites have the lowest thermal conductivity due to the lower carrier thermal conductivity (*κ*_e_). As displayed in Figure 5b, the *κ*_e_ is dominant in the total thermal conductivity (*κ*_tot_). The *κ*_e_ of (Cu_2_Te)_1−x_-(BiCuTeO)*_x_* changes, affected by its electrical conductivity according to Wiedemann law, *κ*_e_ = *LσT*, where *L*, *σ* and *T* are Lorentz coefficient, electrical conductivity and temperature, respectively. Besides, electrical conductivity could be calculated according to *σ* = *neμ*, where *n*, *e* and *μ* are carrier concentration, elementary charge and carrier mobility, respectively. As exhibited in Table 1, from *x* = 0 to *x* = 0.1, the carrier concentration of (Cu_2_Te)_1−x_-(BiCuTeO)*_x_* is decreased, which causes the reduction of the *σ* and further results in the depression of the *κ*_e_. When the content of BiCuTeO changes from 0.1 to 0.2, the carrier mobility is improved a lot, bringing about a high *σ* and further causing the enhancement of *κ*_e_. The reduction of the range from *x* = 0.2 to *x* = 0.4 is owing to the dropping of the *μ*. The *κ*_tot_ decreases to 1.48 W m^−1^ K^−1^ at 623 K with increasing BiCuTeO content, while the thermal conductivity of Cu_2_Te is 2.61 W m^−1^ K^−1^ at 623 K.

**Figure 6:**
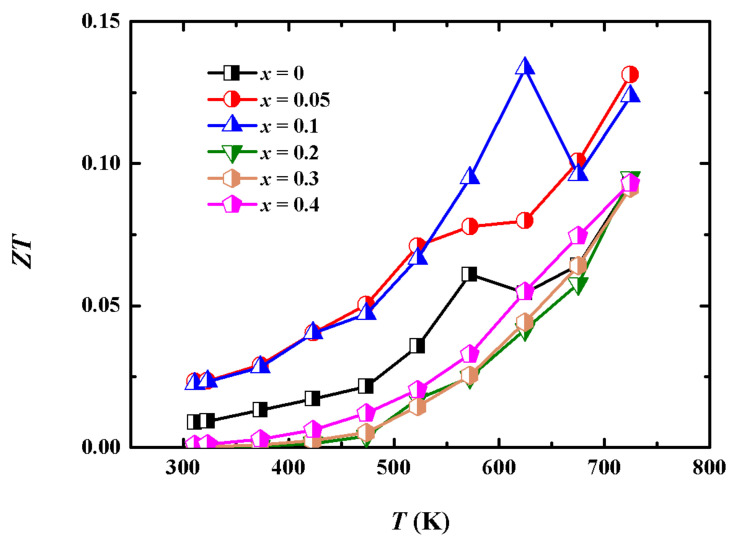
*ZT* of (Cu_2_Te)_1−x_-(BiCuTeO)_x_ as a function of temperature.

Consequently, combining the results of the higher PF and lower *κ*_tot_, the *ZT* values of (Cu_2_Te)_0.95_-(BiCuTeO)_0.05_ and (Cu_2_Te)_0.9_-(BiCuTeO)_0.1_ are higher than that of Cu_2_Te, as illustrated in Figure 6. The *ZT* values of (Cu_2_Te)_0.95_-(BiCuTeO)_0.05_ and (Cu_2_Te)_0.9_-(BiCuTeO)_0.1_ are 0.13 and 0.12 at 723 K, respectively, while the *ZT* value of pristine Cu_2_Te is 0.09 at 723 K.

## 4. Conclusions

In this study, we successfully synthesized (Cu_2_Te)_1−x_-(BiCuTeO)*_x_* composites by SHS combined with SPS. The carrier concentration has been optimized with the incorporation of BiCuTeO, which increased the power factor of (Cu_2_Te)_1−x_-(BiCuTeO)*_x_* and largely decreased carrier thermal conductivity resulting in a lower total thermal conductivity.

Consequently, the (Cu_2_Te)_0.95_-(BiCuTeO)_0.05_ composite sample reached a relatively high *ZT* value of 0.13 at 723 K, which is 41% higher than that of pure Cu_2_Te.

Although the *ZT* of (Cu_2_Te)_1−x_-(BiCuTeO)*_x_* is not high enough, this study lays a foundation for better performance system research.

## Figures and Tables

**Table 1:** Carrier concentration and mobility of (Cu_2_Te)_1−x_-(BiCuTeO)*_x._* (*R*_H_ is Hall coefficient).

Samples	*n* (cm^−3^)	*μ* (cm^2^ V^−1^s^−1^)	*R*_H_ (cm^3^ C^−1^)	m*(*m*_0_)
Cu_2_Te	8.085 × 10^20^	58.58	7.819 × 10^−3^	0.663
(Cu_2_Te)_0.95_-(BiCuTeO)_0.05_	4.459 × 10^20^	54.71	1.400 × 10^−2^	0.785
(Cu_2_Te)_0.9_-(BiCuTeO)_0.1_	3.804 × 10^20^	58.47	1.641 × 10^−2^	0.716
(Cu2Te)_0.8_-(BiCuTeO)_0.2_	1.691 × 10^20^	295.40	3.692 × 10^−2^	0.025
(Cu_2_Te)_0.7_-(BiCuTeO)_0.3_	2.289 × 10^20^	151.50	2.727 × 10^−2^	0.052
(Cu_2_Te)_0.6_-(BiCuTeO)_0.4_	2.379 × 10^20^	102.90	2.627 × 10^−2^	0.110

## Data Availability

Data is contained within the article.
